# A high-throughput sequencing survey characterizing European foulbrood disease and Varroosis in honey bees

**DOI:** 10.1038/s41598-023-28085-2

**Published:** 2023-01-20

**Authors:** Kirk E. Anderson, Duan C. Copeland, Robert J. Erickson, Amy S. Floyd, Patrick C. Maes, Brendon M. Mott

**Affiliations:** 1 Carl Hayden Bee Research Center, USDA Agricultural Research Service, 2000 E. Allen Rd., Tucson, AZ 85719 USA; 2 Department of Entomology and Center for Insect Science, University of Arizona, Tucson, AZ 85721 USA; 3 Department of Microbiology, School of Animal and Comparative Biomedical Sciences, University of Arizona, Tucson, AZ 85721 USA

**Keywords:** Pathogens, Applied microbiology

## Abstract

As essential pollinators of ecosystems and agriculture, honey bees (*Apis mellifera*) are host to a variety of pathogens that result in colony loss. Two highly prevalent larval diseases are European foulbrood (EFB) attributed to the bacterium *Melissococcus plutonius,* and Varroosis wherein larvae can be afflicted by one or more paralytic viruses. Here we used high-throughput sequencing and qPCR to detail microbial succession of larval development from six diseased, and one disease-free apiary. The disease-free larval microbiome revealed a variety of disease-associated bacteria in early larval instars, but later developmental stages were dominated by beneficial symbionts. Microbial succession associated with EFB pathology differed by apiary, characterized by associations with various gram-positive bacteria. At one apiary, diseased larvae were uniquely described as “melting and deflated”, symptoms associated with Varroosis. We found that Acute Bee Paralysis Virus (ABPV) levels were significantly associated with these symptoms, and various gram-negative bacteria became opportunistic in the guts of ABPV afflicted larvae. Perhaps contributing to disease progression, the ABPV associated microbiome was significantly depleted of gram-positive bacteria, a likely result of recent antibiotic application. Our results contribute to the understanding of brood disease diagnosis and treatment, a growing problem for beekeeping and agriculture worldwide.

## Introduction

Honey bees (*Apis mellifera*) are valuable pollinators of agriculture and ecosystems worldwide^1–3^. Recent colony loss has necessitated a review of pollination services and their sustainability including microbiome contributions to health and disease^4,5^. Colony loss is described as multifactorial, often involving combinations of environmental stress, hive management and disease agents^3,6,7^. Strongly associated with colony decline, bacterial diseases of honey bee larvae have become more prevalent worldwide^8–10^. Despite this growing threat, the patterns and processes underlying honey bee larval disease remain poorly understood and poorly diagnosed^11^, with more than half of reported cases in the United States attributed to an unidentified cause.

One primary function of the eukaryotic microbiome is protection from pathogens, and changes to the gut microbiota can range from mildly anti-commensal to pathogenic^12^. Many non-communicable and chronic disease states are associated with microbiome variation, highlighting the importance of microbiome integrity or taxonomic membership in disease susceptibility. A variety of factors may weaken the core microbiota of honey bees rendering the host organism susceptible to disease^5,13^. Ironically, antibiotics used to treat brood disease alter the gut microbiota, often rendering the honey bee more susceptible to disease^14^. The effect of antibiotic application on the larval microbiome is little known, but is predicted to result in an overall decrease of gram-positive bacteria throughout the network of social nutrient processing. The healthy larval microbiome is comprised primarily of native commensal species including *Bombella apis* and *Apilactobacillus kunkeei,* previously referred to as *Parasaccharibacter apium and Lactobacillus kunkeei*^15^. Closely allied with nutrient processing, these two oxygen tolerant species also dominate honey and beebread, worker crops and hypopharyngeal glands, queen and worker mouthparts and the anterior alimentary tract of queens^16^. Both bacterial species are host co-evolved, associated with decreased abundance of honey bee-specific disease, and likely represent protection from various opportunists including bacteria, microsporidia, and fungi, omnipresent throughout social resource space^5,17–20^. The influence of the microbiome on host fitness highlights the role of commensals in mediating disease susceptibility^12,13,21,22^.

Two very different bacterial diseases afflict honey bee larvae: American foulbrood (AFB) caused by *Paenibacillus larvae,* and European foulbrood (EFB), attributed to *Melissococcus plutonius*^23–25^. While AFB disease is overt, highly virulent, and caused by a singular bacterial species, a field diagnosis based on EFB-associated symptomology is often discordant with subsequent lab tests, revealing a lack of *M. plutonius*. Historically, EFB has been considered an opportunistic disease, affecting stressed hives associated with particular crops, seasons or environmental condition^24,26^. Additionally, *Melissococcus plutonius* strains possess widely different virulence genes associated with disease progression^27^. Collectively, these findings suggest that *M. plutonius* is both a typical hive inhabitant that can cause or contribute to disease under stressed conditions, and a highly communicable and virulent disease according to the presence/absence of virulence genes^27–32^.

Although considered the primary cause of EFB disease, *M. plutonius* often goes undetected when EFB-like symptoms are present in larvae^33^. Larval disease with symptoms similar to EFB, but lacking confirmation of *M. plutonius* was first described as parasitic mite syndrome, because it occurred with long-term infestations of the ectoparasitic mite *V. destructor*^34^. The disease was later named Varroosis because the *V. destructor* mite was considered primary for the transmission of secondary viral infections^35,36^. However, the larval symptoms can also occur in the absence of *V. destructor* infestation, requiring a different namesake; Idiopathic Brood Disease Syndrome (IBDS)^37^. Both Varroosis (PMS) and IBDS are diagnosed by the presence of brood at different ages that appear molten (melted) on the bottom of cells or a collection of symptoms similar to, but not matching EFB, AFB or “sac brood”, a virus known to afflict larvae^38^. It is hypothesized that a virus is associated with melty larval symptomology, and the level of *V. destructor* infestation may then accelerate viral transmission and/or replication^34,37^. *V. destructor* is a demonstrated viral vector^38,39^, and both viral function and parasitism by *V. destructor* can suppress the immune system of adults or larvae leading to accelerated disease^40,41^.

**Figure 1:**
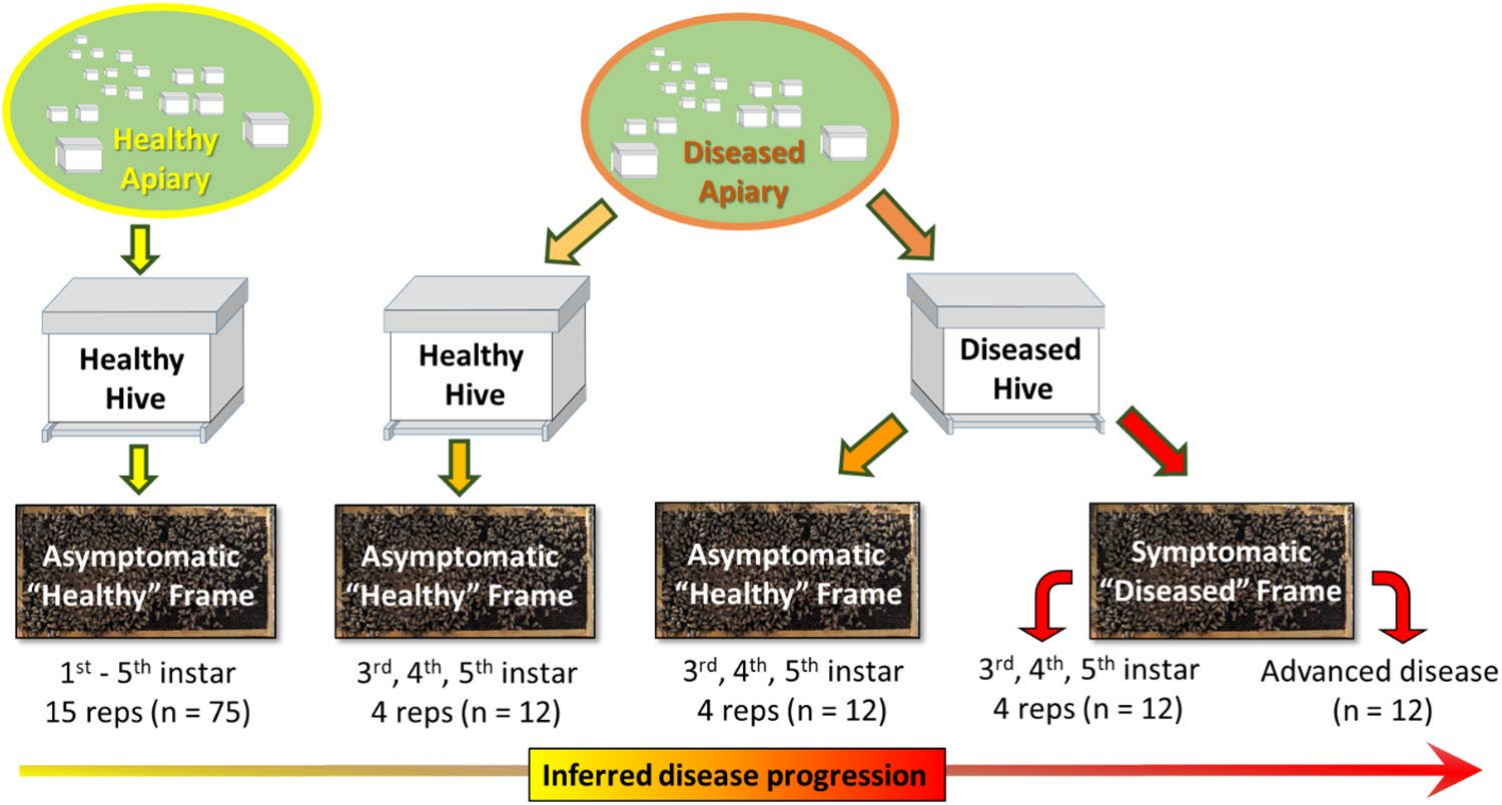
Experimental design and sample size used to infer disease progression. From a disease free “Healthy” apiary in Tucson AZ, we sampled first through fifth instar larvae from three different colonies, five repetitions per colony. From each diseased apiary in Illinois, we sampled one “Healthy” hive with no disease symptoms, and one “Diseased” hive. From the diseased hive, we sampled both asymptomatic and symptomatic larvae. We sampled 3rd, 4th, and 5th instar larvae and also advanced disease wherein larvae instar was indeterminate.

In this study, we explore the contribution of the microbiota to disease progression. We analyzed the microbiota throughout larval development from seven different apiaries; one with no recent history of EFB disease, and six apiaries with active brood disease comprised of both symptomatic and asymptomatic colonies. We used qPCR to quantify virus levels, and estimate bacterial abundance, and high-throughput sequencing to explore the bacterial microbiota associated with both healthy and disease symptomology. We sequenced young, middle, and old aged larvae (3rd, 4th and 5th instars) to characterize shifts in disease progression according to age, development and proximity to disease symptoms (Fig. 1). To discover bacteria that may contribute to pathology, we sampled asymptomatic larval phenotypes from both symptomatic and asymptomatic colonies. We also sampled larvae with advanced disease symptoms to define bacterial species associated with the final stages of disease.

## Methods

### Disease diagnosis

Larval disease in the field is diagnosed by changes to healthy larval or pupal morphology, herein referred to as symptomology. The cause of disease can then be confirmed to differing degrees based on lab or field tests including staining and microscopy, qPCR, next generation sequencing, or lateral flow device^6,9^. During the first three days of larval development, adult nurse workers feed larvae a highly nutritious and antimicrobial jelly secreted from special head (hypopharyngeal) glands. Larvae gain weight rapidly after the third instar, but most of the weight is gained as a fifth instar larva, which generally fills out the bottom of its cell^42^. Healthy larvae remain pearly white and plump, floating in their own food when young, on their side with spiracles up, curled symmetrically in a “C” shape within the wax cell (File S1). Larvae require 5–6 days to develop from egg hatching to the onset of pupation when they turn vertical within their cell and spin a cocoon, then adult workers seal their wax cell, called “capping”.

Symptoms can be affected by colony status, and differ by larval instar^33^. When infected with *Melissococcus plutonius,* younger larvae become flattened and/or displaced within their cell, while larger, more acutely infected larvae become flaccid and turn from white to yellow to brown, as their gut is compromised and their tissue loses integrity^24^. Often larvae become translucent with their internal tracheal network more visible through the thin cuticle. In the final stages of disease, larvae dehydrate and form a dark brown scale, easily removed from the wax cell. Individual bee larvae usually die of EFB disease as fourth or fifth instars, 1–2 days before pupation^24^.

A variety of larval disease symptoms have been reported that do not match any known cause. These disease states are also distinguished by changes in shape, position and color atypical of healthy larvae. In colonies suffering from PMS (Varroosis) or IBDS, larvae of different ages appear molten at the bottom of their cells, accompanied by odors that differ from EFB and AFB^34,37^. Considering the progression of disease, symptoms of Varroosis/IBDS can resemble EFB, but color changes and odors associated with PMS larvae are attributed to age, decomposition or secondary bacterial opportunism. Here we photographed larvae at various stages of development to quantify the change in symptomology associated with disease states and secondary invaders (File S1). We photographed colonies with variable resolution, to examine both individual larva and brood frame character. At the colony-level, a diseased brood frame shows a spotty (shotgun) pattern due to the hygienic behavior of adults removing diseased brood^24^. We examined the relationship between symptomology and molecular data using a test of proportions.

### Sampling and experimental design

As an apiary control for active disease, we sequenced the microbiomes associated with larval development from a disease (symptom) free apiary in Tucson AZ, with no history or recent manifestation of bacterial brood disease (Table 1, File S2). We confined three separate mated queens over empty wax comb overnight (21 h) to generate a cohort of relatively same-age eggs. Few or no eggs are produced in the first 6 h, and eggs required 75–80 h to hatch. When approximately 50% of the eggs had hatched, we sampled five larvae from each queen every 24 h; when larvae were an estimated age of 8, 32, 56, 80, and 104-h-old (± 8 h), roughly corresponding to early first through late fourth instar larvae. Larvae sampled from each time period were weighed to examine the change in microbiome size and character relative to larval mass.

**Table 1:** Metadata associated with larval sampling.

Apiary ID	Date Sampled	Latitude	Longitude	Sample size	Disease state^a^
Tucson, AZ	May 1–5, 2017	32.2610	− 111.0078	75	None
Spring Valley, IL	June 22, 2016	39.8805	− 90.9754	47	EFB
Butler, IL	July 8, 2016	39.1834	− 98.5434	48	EFB
Hardin, IL	Sept. 6, 2016	39.1663	− 90.6189	50	EFB
Kampsville, IL	Sept. 15, 2016	39.2964	− 90.6114	48	EFB
Wright's Corner, IL	Oct. 15, 2016	39.1176	− 88.9024	32	EFB
Hull, IL	Oct. 8, 2016	39.7559	− 91.2050	48	PMS/ABPV

^a^EFB is European foulbrood, and PMS/ABPV is parasitic mite syndrome /acute bee paralysis virus.

To explore the microbial succession of diseased larvae, we employed an Illinois State apiarist to perform a survey of brood disease in Illinois, sampling disease states identified in the field as EFB or EFB-like according to larval symptomology detailed above (Table 1). From each apiary we sampled both healthy and diseased colonies, and an age range (3rd, 4th and 5th instar) of symptomatic and asymptomatic larvae to infer disease progression (Fig. 1). We collected N = 48 larvae per apiary, sampling four replicates for each of three larval stages from two hives per apiary (one healthy, one diseased). We sampled one healthy frame from a healthy hive and both a healthy and diseased frame from a diseased (symptomatic) hive. Thus our sampling produced n = 12 asymptomatic larvae from a symptom free hive, n = 12 asymptomatic larvae from a diseased colony, n = 12 symptomatic larvae from the diseased colony with early disease, and n = 12 larvae from the diseased colony (unknown larval instar) with advanced disease.

### Nucleic-acid extraction

Larvae were individually collected in RNAlater solution (Ambion #AM7021) per manufacturer’s protocol, frozen at − 20 °C for < 6 months, then frozen at − 80 °C until nucleic-acid extraction via Mo-Bio PowerViral™ Environmental RNA/DNA Isolation Kit (#28000-50). Briefly: individual larvae or larval material were placed into a 2 ml bead-beating tube containing ~ 100 µl of 0.1 mm silica-zirconia beads and 600 µl of solution PV1/βME, then homogenized for a total of 2 min in 30 s intervals via BioSpec Mini-Beadbeater-16. Total RNA and DNA were obtained from the bead-beaten homogenates following the standard PowerViral kit protocol. DNA fractions for each sample were used for 16S rDNA amplicon sequencing and bacterial quantification via qPCR. RNA fractions were used to create cDNA for virus-screening via real-time quantitative-PCR (RTqPCR).

### Total bacterial quantification

We quantified total bacterial abundance in larvae with a real-time PCR (qPCR) assay quantifying 16S rRNA gene copies^40^. We first generated a standard curve using a tenfold serial dilution of a plasmid standard containing a full-length *Escherichia coli* 16S rRNA gene. We than amplified a 466 bp fragment in the V3–V4 region of the bacterial rRNA gene from a pool of total DNA using universal (degenerate) primer pair (5′-CCTACGGGDGGCWGCA-3′ and 5′-GGACTACHVGGGTMTCTAATC-3′). Quantitative PCRs were performed on a BioRad CFX96 thermocycler in 12 μl reactions containing 9 μl of iTaq Universal SYBR Green Supermix (BioRad), 0.5 μl forward primer, 0.5 μl reverse primer, and 2 μl of DNA template. The cycling conditions were 95 °C for 3 min followed by 40 cycles of 95 °C for 10 s and 60 °C for 60 s. The qPCR results were expressed as the total number of 16S rRNA gene copies per DNA extraction (100 μl volume elution). Bacterial copy number comparisons were made using one-way ANOVA (Tukey HSD post-hoc) and two-sample t-tests.

### PCR and MiSeq

To characterize the microbial communities associated with larval disease, we amplified the V3–V4 region of the 16S rRNA gene using PCR primers (341F 5′-CCTACGGGNGGCWGCAG-3′; 805R 5′-GACTACHVGGGTATCTAATCC-3′). Similar to a previous publication by the same authors^6^ amplification was performed using the HotStarTaq Plus Master Mix Kit (Qiagen, USA) under the following conditions: 94 °C for 3 min, followed by 28 cycles of 94 °C for 30 s, 53 °C for 40 s and 72 °C for 1 min, with a final elongation step at 72 °C for 5 min. PCR products were confirmed using a 2% agarose gel. PCR products were then used to prepare DNA libraries following Illumina MiSeq DNA library preparation protocol. Sequencing was performed at the University of Arizona Genetics Core (UAGC) on a MiSeq following the manufacturer’s guidelines. All sequence data were deposited in GenBank under Bioproject ID: PRJNA897937.

### 16S rRNA gene community analysis

16S rRNA gene sequences were processed using MOTHUR v.1.44.3^43^ according to previously published protocols^6^. Briefly, paired end reads were joined using the make.contigs command. After the reads were joined, we removed the first and last five nucleotides using the SED command in UNIX. Sequences were screened to remove ambiguous bases, using the screen.seqs command. Unique sequences were generated using the unique.seqs command. A count file containing group information was generated using the count.seqs command. Sequences were aligned to BEExact database^44^ using the align.seqs command. Sequences were filtered to remove overhands at both ends and gaps using filter.seqs. The unique.seqs command was ran again to remove new redundancies from filtering. A precluster step using pre.cluster was performed. Chimeras were removed using chimera.uchime command^45^. Sequences that were not bacterial in origin were removed using the remove.seqs command. All unique sequences with one or two members (single/doubletons) were removed using the AWK command in UNIX. A distance matrix was constructed for the aligned sequences using the dist.seqs command. Sequences were classified at the unique level with the BEExact database using classify.seqs command. Unique sequences were then merged at the species-level with the merge.otus command.

Data were curated to remove sources of contamination associated with low abundance DNA environments^46,47^. Samples of first and second instar larvae contain very little DNA and these results were used to guide our selection criteria. Three exclusion criteria were used to identify contaminant OTUs: (1) Significant and strong negative correlation of read number with microbiome size (Pearsons coefficient > 0.50), (2) Intercorrelations of known contaminants with suspect contaminants, and (3) An assessment of OTU/host association including honey bees and well-curated culture collections associated with soils, water, humans, laboratory, and demonstrated “kitome” contamination.

### Statistical analyses

Microbiome data sets employing universal primer sets are compositional, violating a number of assumptions required for parametric analysis^48^. To construct PCAs^49^ or perform parametric analyses, we first converted bacterial relative abundance to ratios among all operational taxonomic units (OTUs)^48^ using the software CoDaPack’s centered log-ratio (CLR) transformation^50^. Following data curation, retained OTUs were normalized by qPCR BactQuant values by first calculating the proportion of each OTU relative to the total number of retained sequences produced with Illumina sequencing. Each OTU ratio was then multiplied by 16S rRNA gene copies quantified from qPCR. Next, each OTU was corrected for 16S gene copies per bacterial cell based on their closest taxonomic representative^51^. Those OTUs lacking a close taxonomic representative in the database were assigned 4.2 gene copies, the mean 16S rRNA gene copy number across all known bacteria^52^. Next, the data were CLR-transformed and these values were used to perform principle component analysis (PCA) plotting bacterial community composition for each apiary site and condition. We compared microbial community structure with PermANOVA and ANOSIM comparing Bray–Curtis similarity and Jaccard values by apiary, larval instar and disease symptomology. A Wilcoxon Rank-sum test was used to examine the absolute abundance of each OTU by apiary and disease status. We performed correlations examining log transformed bacterial abundance for each major bacterial taxon. All analyses were conducted in either JMP_v11 (JMP_1989–2007) and/or SAS_ v9.4^53^.

### Real-time qPCR of virus

Three of the Illinois sites (Spring Valley, Butler, and Hull) were screened via real-time qPCR to determine the prevalence and abundance of three paralytic viruses associated with parasitic mite syndrome: Acute Bee Paralysis Virus (ABPV), Deformed Wing Virus (DWV), and Kashmir Bee Virus (KBV). Template cDNA was generated as follows: 8 μl of extracted RNA/DNA was treated with DNase I (1 μl enzyme + 1 μl buffer, Ambion #AM2224), then the whole 10 μl reaction was used as template in a 20 μl cDNA synthesis per manufacturer’s protocol (RevertAid First Strand cDNA Synthesis Kit, ThermoScientific #K1622). The 20 μl cDNA reaction was then diluted with 180 μl of nuclease-free water prior to qPCR.

Real-time qPCRs were performed as follows: 95 °C for 5 min, then 45 cycles with 94 °C for 20 s and 60 °C for 30 s, followed by a high-resolution melt curve. Reactions utilized Luna Universal qPCR Master Mix (NEB #M3003E) in triplicate on a CFX96 Real-Time PCR Detection System (Bio-Rad). Each 12 μl reaction contained 6 μl Luna mix, 0.5 μl of forward and reverse primers (10 μM), 2 μl of cDNA template, and 3 μl H_2_O. We used the following primer sets in the reactions DWV^54^: F-5′-ATTGTGCCAGATTGGACTAC-3′, R-5′-AGATGCAATGGAGGATACAG-3′, KBV^55^: F-5′-ATGACGATGATGAGTTCAAG-3′, R-5′-AATTGCAAGACCTGCATC-3′, and ABPV^56^: F-5′-AATGGGCCTATGGACTTTTCTA-3′, R-5′-AAATCTCCTGCAATAACCTTGG-3′.

To confirm the absence of contaminant DNA and primer dimers, no-template controls (consisting of reaction mix and water) and melt-curve analyses were included on each qPCR plate. Relative viral abundance was estimated via 2–∆∆Ct method^57^ including two honey bee mRNA reference genes (β-actin and RPS18), and all values were then expressed relative to mean viral load in the asymptomatic, presumably healthy, Spring Valley colony (n = 12). For statistical purposes, we assigned a Ct value of 40 to samples with null quantification. Finally, we log-transformed relative viral abundance to approximate normality and tested for differences in viral abundance by apiary and symptomatic sample-type using both parametric (ANOVA plus Tukey’s HSD) and non-parametric (Kruskal–Wallis plus Steel–Dwass) tests in JMP 14.

## Results

### Larval microbiota from an asymptomatic apiary

**Figure 2:**
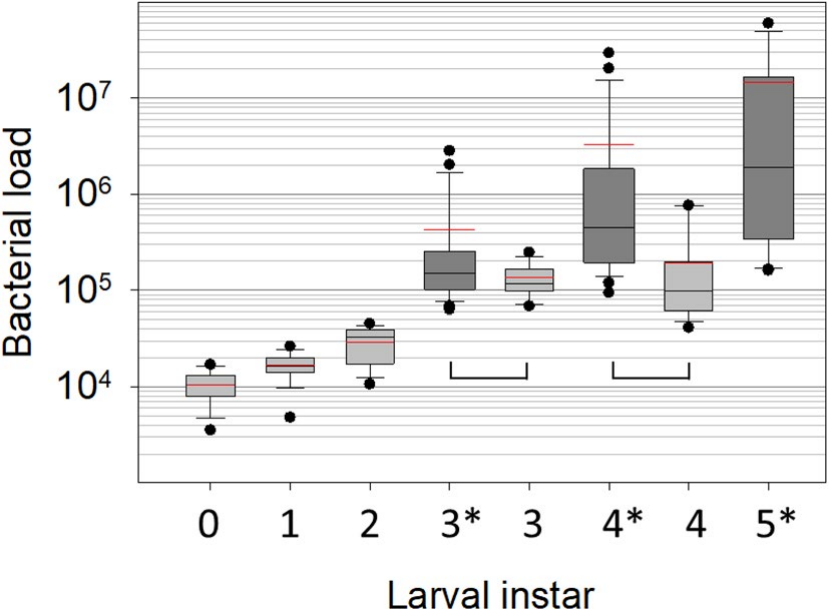
Bacterial load (log scale) of asymptomatic larval instars differs by apiary. Boxplots contain 25–75% of the data, whiskers are 10th and 90th percentiles, and dots represent the range. The red and black horizontal lines represent the mean and median respectively. Light grey box-plots represent larval instars 0–4 from an apiary in Arizona with no history or recent evidence of EFB disease (n = 15 for each larval instar, see Fig. 3). The dark grey box-plots* represent samples of asymptomatic larvae from asymptomatic colonies in apiaries in Illinois with active EFB disease (n = 23 or 24 for each larval instar). As indicated by brackets above the x-axis, asymptomatic larval instars differ in bacterial load according to t-tests of log-transformed normalized abundance (3rd instar; T36 = − 1.8 p < 0.07, 4th instar; T36 = − 3.9, p < 0.0004).

**Figure 3:**
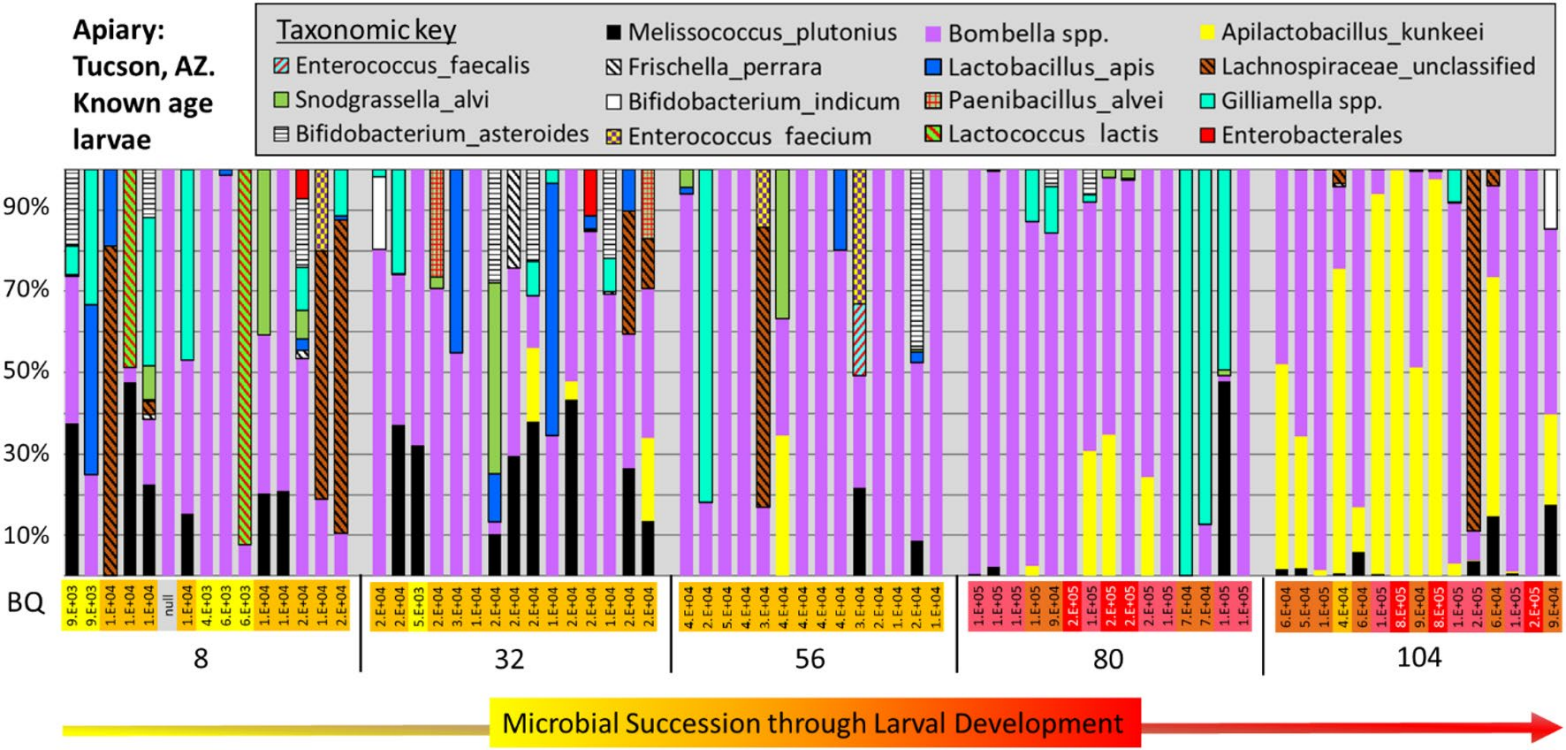
Microbial succession associated with healthy larval development sampling each 24 h to approximate zero to fourth instar. Results from an apiary in Tucson, AZ. with no history or recent evidence of European foulbrood disease. Data is from three colonies, five reps per day per colony, yielding n = 15 for each larval age (x-axis). Estimated average hours after egg hatch (range =  ± 8 h) is listed across the x-axis. Individual larval microbiotas are displayed as relative abundance bar charts (y-axis), and BactQuant (BQ) values estimating microbiome size are displayed as a log notation heatmap vertically across the x-axis.

To provide a disease free comparison for apiaries with active brood disease, we sequenced the microbiomes of larvae from an asymptomatic apiary in Tucson, AZ, with no history of bacterial brood disease. Mean larval mass for 8, 32, 56, 80, and 104-h-old larvae was 1.6, 1.7, 4.5, 11.7, and 42.3 mg. respectively. The mean bacterial load of known age healthy larvae was 10^5^ gene copies, ranging from 4 × 10^3^ to 8 × 10^5^ (Fig. 2). Eight and 32-h-old larvae were at the limits of detection for qPCR averaging 10^4^ copies. Across early first (8 h. old) to fourth instars (104 h. old), *Bombella* spp. occurred with the greatest prevalence and abundance followed by *A. kunkeei* and *M. plutonius* (Fig. 3). Consistent with larval size and feeding intensity, 80 and 104-h-old larvae had significantly larger microbiotas (10^5^) dominated by *Bombella*, *A. kunkeei* and *Gilliamella*. Also considered a bacteria that co-occurs with EFB disease, *Apolactobacillus kunkeei* (formerly *Achromobacter eurydice*^58^) became more abundant in late instars, while *Lactobacillus apis* was more abundant in early instars. Consistent with past culturing results^59^, core hindgut bacteria of adult workers including *Gilliamella, Lactobacillus, Snodgrassella, Frischella* and *Bifidobacterium*, all occurred in early instar larvae. The first three larval instars were host to a sporadic variety of species known to co-occur with, or even cause larval disease, including *M. plutonius, Paenibacillus alvi,* and *Enterococcus faecalis*^24,29,60^. Prevalent in larvae from the genus *Apis*^61,62^, an undescribed species of Lachnospiraceae occurred with sporadic abundance in the asymptomatic Arizona apiary and at five of the six Illinois apiaries with active disease.

### Disease diagnosis

Consistent with our experimental design targeting third, fourth and fifth instar larvae (Fig. 1), the Illinois state apiary inspector provided detailed photographs and various verbal descriptors of disease symptoms in larvae (File S1). In general, the close-up photographs of symptomatic larvae were highly consistent with disease descriptions. The five apiaries with EFB disease presented typical and well-known symptomology associated with *M. plutoniu*s disease progression, including rapid desiccation, color changes indicative of oxidation (off white), proceeding from yellow to brown, and the collapse of internal cellular structure associated with advanced disease finally resulting in a scale^24^. Listed by frequency of use from high to low, the EFB disease states were described as oxidized, twisted, brown, having lost definition, flattening, displaced, and yellow. Other less frequently used descriptors with a unique meaning were scaly, scabby, spotty and bruised, and terms related to putrification like liquid, puddle and gooey (File S1).

The significant relationship between symptomology and molecular results reveals two very different disease states; European foulbrood at five of the six Illinois apiaries, and putative parasitic mite syndrome (Varroosis) at the Hull apiary. A cluster analysis of words used to describe disease symptoms indicates significantly different symptomology by disease type wherein the term “melting” was unique to the Hull apiary and used many times to describe diseased larvae (File S1). Other descriptors unique to the Hull apiary were “deflated”, “dull”, and “*V. destructor* mite”. Based on a test for proportions, the “melting and deflated” terminology at the Hull apiary was significantly associated with estimated copy number of ABPV > 10^7^, averaging > 10^9^ (p < 0.0001).

From the apiaries with EFB disease, 84% of larvae designated as symptomatic by the apiary inspector had disease microbiomes dominated by *M. plutonius*. The remainder were dominated by *Enterococcus faecalis*, *Frischella perrara*, and *Bombella* spp. Within an EFB positive colony, the microbiota of asymptomatic larvae was dominated by *M. plutonius* in 35 of 60 individuals at > 50% relative abundance. This was a significantly greater proportion (p < 0.001) than that recorded for the EFB negative colonies from the same EFB positive apiaries (10 of 60).

### Sequence read curation

Next generation sequencing returned 16,769, 830 raw reads (400 bp) for the 348 libraries, approximately 48 libraries per disease apiary, and 75 libraries dedicated to the characterization of known age larvae (Table 1, File S2). Associated with high‐throughput metagenomic studies, laboratory contamination can be linked to reagents, laboratory facilities, extraction methods, researcher and extraction year^46,47^. First instar larvae represent a honey bee environment with little to no microbial DNA abundance (1000 s of gene copies) and bacterial load and diversity is known to increase with larval development and mass^59,63^. The taxonomy returned for first and second instar larvae reinforced the hypothesized pattern of contamination as determined by our three criteria. Across the total sequence read data set (n = 345), many OTUs were highly inversely correlated with microbiome size (r-square values), and with one another (File S2). Much of the taxonomy returned for these suspect OTUs was associated with various environmental sources, not order Hymenoptera or the pollination environment^44^. Based on results from the combined criteria we designated the following OTUs as the top eight contaminants: *Ralstonia, Caulobacter, Bradyrhizobium, Pelomona*s, Cyanobacteria, *Lysinibacillus, Shigella*, and *Nevskia*, and more generally, Chitinophagaceae, Comamonadaceae, Caulobacteraceae, Burkholderiaceae, and Bradyrhizobiaceae. Our results linking particular OTUs to contamination is reinforced by previous results sequencing blank controls or low abundance honey bee environments like the queen mouth and midgut^64^. From that study (see highlighted taxa in Table S12, Queen microbiota correlations), at least six major OTUs were significantly associated with the low abundance environments including many of the same contaminant OTUs implicated here.

Following curation of the 16S rRNA gene amplicon data set, we retained 10,662,664 quality trimmed reads, an average of 30,640 per library (File S2). Although we applied rather strict criteria to remove contaminant sequences, we entertain the possibility that some of the low prevalence/abundance OTUs may be symbionts. Although these questionable contaminant OTUs don’t alter our primary results, many are considered common lab contaminants and should be interpreted with caution. These suspect OTUs were characterized by a much lower correlation with microbiome size and other designated contaminants, a hybrid occurrence pattern between contaminant and confirmed symbiont (File S2), and an association with honey bees in the past based on culturing, including *Bacillus*, *Delftia*, *Acinetobacter, Pseudomonas, Sphingomonas, Streptococcus, and Staphylococcus* among others^65–67^.

### Microbial community analysis

The BEExact classifier placed many of the top OTUs to species level^44^. From the diseased apiaries, eight OTUs contributed the majority of variation to changes in relative and absolute abundance accounting for 90% of the sequence total in the curated data set, from most to least abundant; *M. plutonius, Bo. apis, A. kunkeei, E. faecalis, F. perrara, Lachnospiraceae, F. fructosus* and *L. apis* (File S2). The top 17 OTUs accounted for 95% of the curated sequence total but many of the top OTUs had greater than 50% sparsity throughout the data set rendering MANOVA analyses uninformative. Following classification and data curation, we analyzed the top 55 OTUs with Permanova and PCA, and performed Wilcoxon analyses comparing the top 17 OTUs by apiary, larval instar and disease symptomology (File S3).

**Figure 4:**
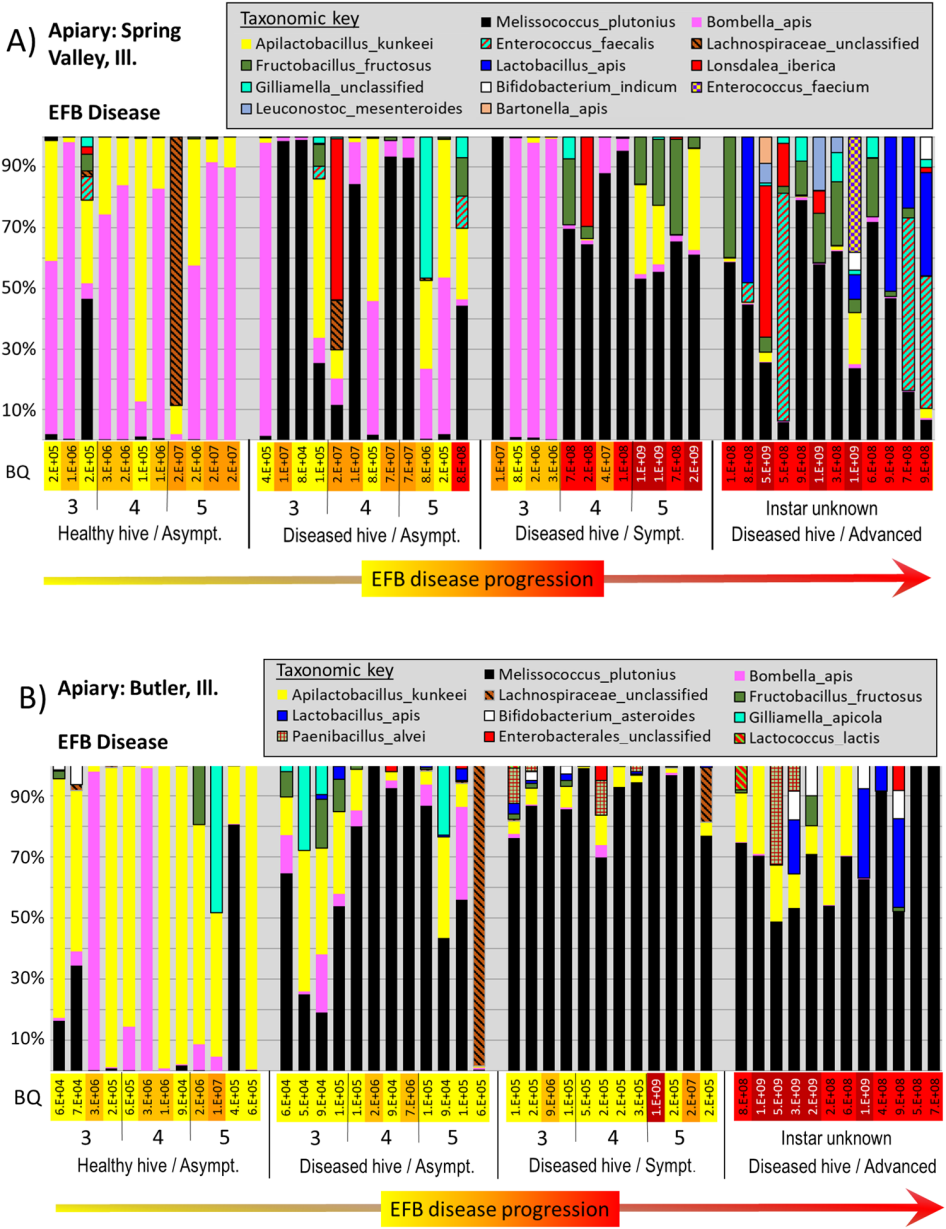

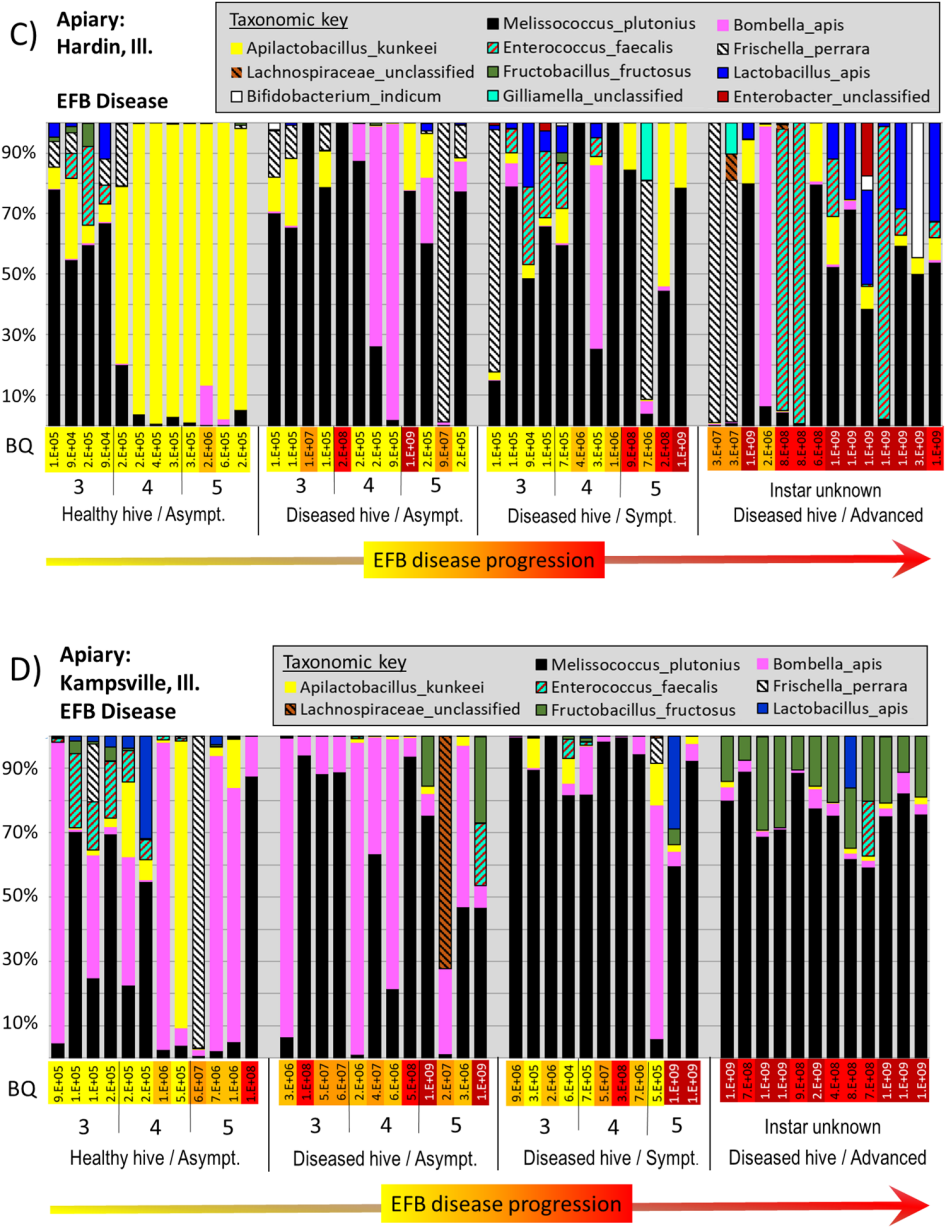

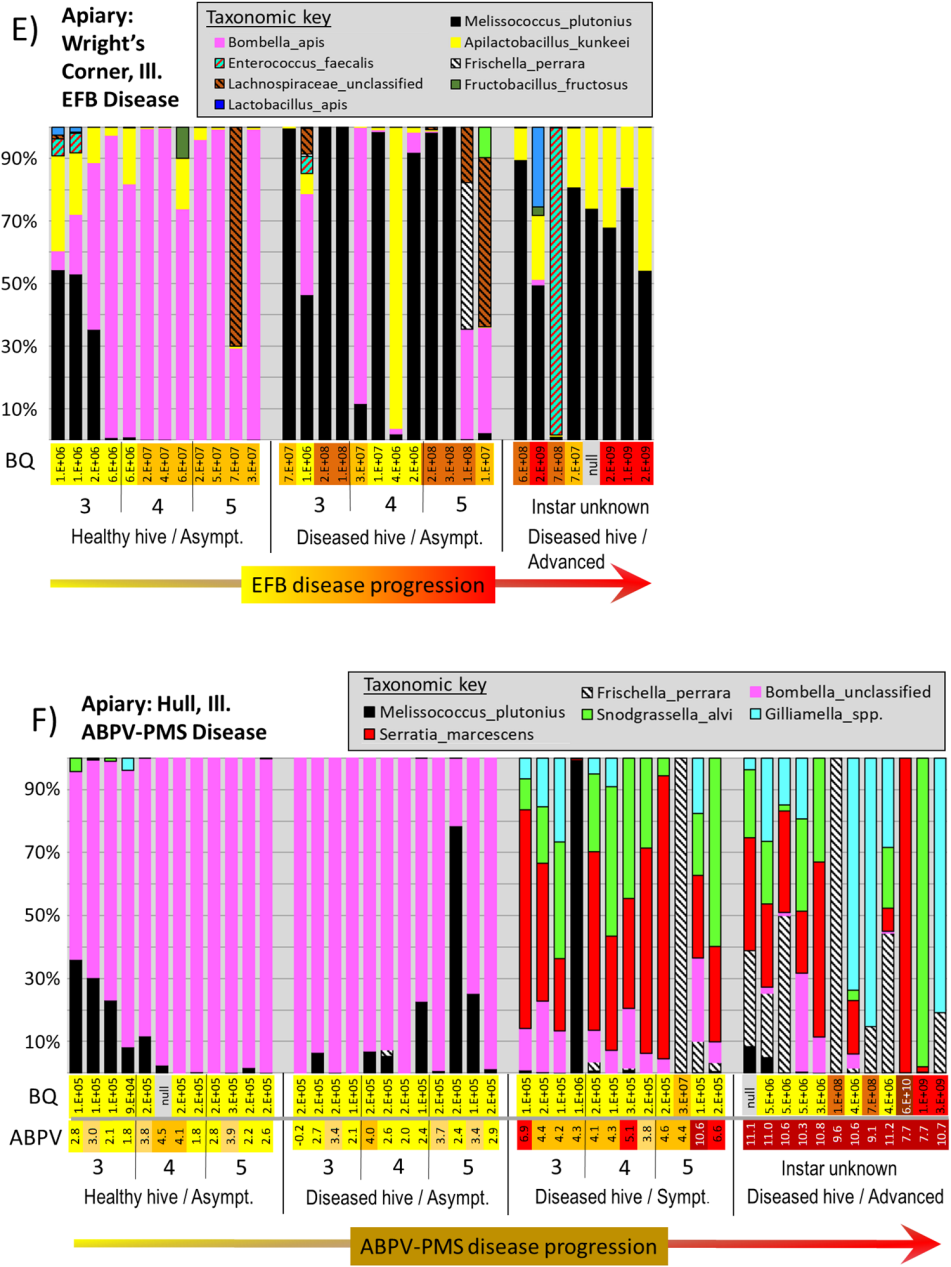
Panels (**A**–**F**) illustrate disease progression of the larval microbiota from six different apiaries; five with European foulbrood disease (**A**–**E**) and one with Acute bee paralysis virus-Parasitic mite syndrome (panel **F**). Each vertical bar represents a single larval microbiota displayed as relative abundance (y-axis). Larval instar and symptomology are displayed across the x-axis; from left to right are four distinct sample groups selected to capture disease progression: Asymptomatic larvae from an asymptomatic colony, asymptomatic larvae from a symptomatic colony, symptomatic larvae from a symptomatic colony, and advanced disease from a symptomatic colony. BactQuant (BQ) results estimating microbiome size are listed as log notation vertically across each x-axis. On panel (**F**), an RT-qPCR estimate of ABPV viral load is shown at log10 scale below BQ values.

Within the five apiaries diagnosed with active EFB disease, sequences matching *M. plutonius* were present in 95% of asymptomatic larvae from asymptomatic (healthy) hives, at an average relative abundance of 6% (range 0–92%) across the 71 asymptomatic larvae (Fig. 4A–E). In contrast, the prevalence of *M. plutonius* in asymptomatic larvae (56, 80 and 104 h old) from the disease free apiary in Tucson was significantly less at 40% (Z = 6.6 p =  < 0.00001), with significantly lower mean relative abundance at 2.4% (range 0–16%).

In general, bacterial abundance at the EFB apiaries increased with larval mass and disease progression (Fig. 4A–E), and total bacterial abundance was positively correlated with the abundance of *M. plutonius* (R-sq = 0.51, p < 0.0001). We found a significant difference in total bacterial abundance between healthy larval instars by age, between asymptomatic larval instars and those with early symptoms of disease, and between larvae with early and advanced symptoms (File S3). Microbiota size was primarily, but not entirely driven by the presence of *M. plutonius.* Other bacteria were sporadically associated with high bacterial loads in symptomatic larvae (> 10^8^), including *Enterococcus faecalis, Frischella perrara, Gilliamella spp. and Serratia marcescens.* Bacterial diversity increased significantly in the presence of disease (File S3), all but one diseased apiary (Wright’s Corner) had significantly increased number of observed species than did known age larvae sampled from a disease free apiary.

### EFB microbiota differs by apiary

A principle components analysis (PCA) reveals significantly different groupings according to EFB apiary and larval symptomology (File S3). Based on PermANOVA, larval microbiomes differed by apiary; F = 13.7; R-sq: 0.20; p < 0.001, instar (age); F = 4.2; R-sq = 0.04; p < 0.001, and symptomology at the colony; F = 27.3; R-sq = 0.09, p < 0.001, and individual level; F = 19.4; R-sq = 0.07, p < 0.001. The Hull apiary clustered independently from the EFB diseased apiaries (File S3). EFB was defined by *M. plutonius* dominance, but the Hull apiary samples contained a low abundance of *M. plutonius* regardless of larval instar, colony disease status or symptomology, and contained a unique assemblage of bacterial OTUs not found at the other apiaries (File S3). Thus, we performed statistical analysis on the microbiomes of EFB disease separately from those of the Hull apiary.

*M. plutonius* prevalence and abundance differed significantly by apiary, by asymptomatic and symptomatic colony, and when comparing early and advanced disease (File S3). Based on Wilcoxon analysis, *M. plutonius* was more abundant at Kampsville and Hardin apiaries. Hardin contained the most *A. kunkeei*, while Kampsville contained the most *Bo apis*. Associated with small microbiota size, *Bo apis* and *L. kunkeei* were generally abundant in asymptomatic larvae (Fig. 3). In general, *Bo. apis* was not associated with advanced disease, and decreased with both microbiome size and EFB disease progression. In contrast, various Firmicutes were significantly associated with early or advanced disease states by apiary (Fig. 4A–E, p < 0.001). *M. plutonius* occurred with different taxa by sampled apiary (Fig. 4, File S3). For example, *Enterococcus faecalis* was more abundant at Spring Valley and Hardin, *F. fructosus* was more abundant at Kampsville and Spring Valley, while *A. kunkeei* was more abundant at Hardin, Butler and Wright’s Corner (File S3, Fig. 4A–E).

The apiary diagnosed as parasitic mite syndrome (PMS) was analyzed separately (File S3). At this site, only samples classified as advanced disease showed increased microbiome size. Three bacteria associated with the adult worker ileum (*F. perrara, G. apicola* and *S. alvi*) were significantly more abundant in larvae with putative PMS, as was *Serratia marcescens* (File S3). All four of these bacteria attained abundance > 10^8^ in separate larvae with advanced disease (Fig. 4F). Additionally, the abundance of *S. alvi* was positively correlated with that of *S. marcescens* (Fig. 4). Associated with smaller microbiome size (2 × 10^5^), *Bombella apis* dominated healthy larval phenotypes sampled from the Hull apiary.

### Real-time qPCR of virus

**Figure 5:**
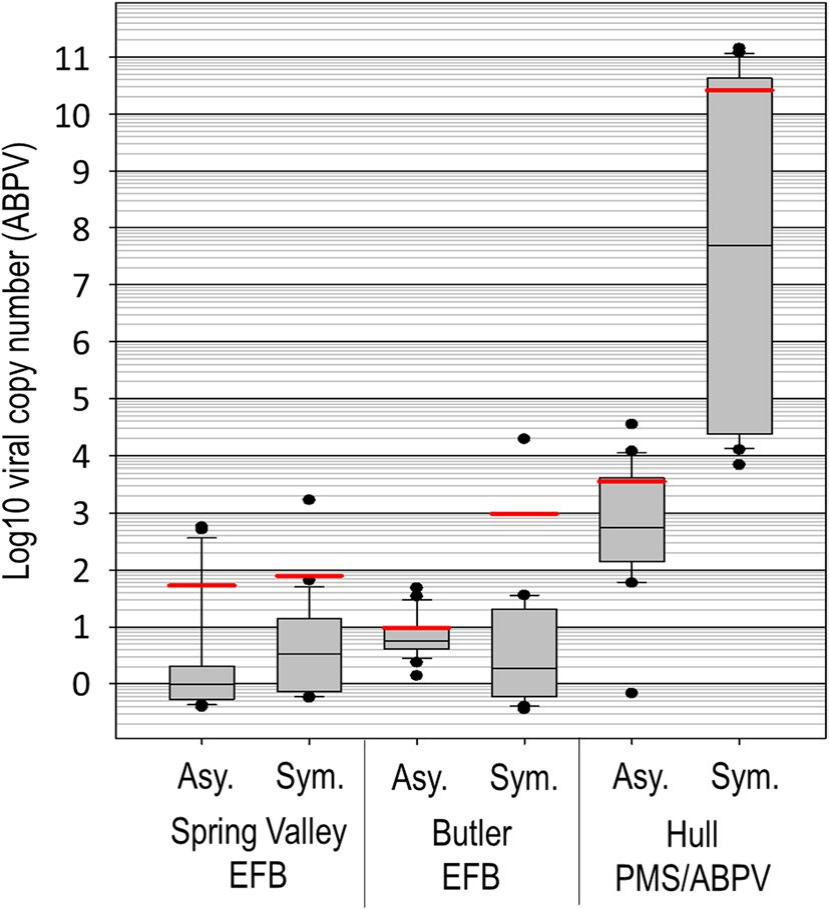
Estimated copy number of acute bee paralysis virus (ABPV) in symptomatic (Sym.) and asymptomatic (Asy.) larvae diagnosed with either European foulbrood (EFB) disease or parasitic mite syndrome (PMS). All displayed values are relative to the geometric mean viral load of asymptomatic larvae from the asymptomatic Spring Valley colony. Each boxplot is comprised of 20–24 individual larvae (see File S4 for details). The grey box contains 25–75% of the data, whiskers are 10th and 90th percentiles, and dots represent the range. The red and black horizontal lines represent the arithmetic mean and median respectively.

Based on the microbiome results, and our word-specific analysis assigning values to various symptoms, the disease state at the Hull apiary was not EFB or AFB, and was unlikely caused by bacteria. Based on the frequency of *V. destructor* mites detected in the Hull apiary photographs (File S1), and the appearance of brood in general, we hypothesized that the larvae may be afflicted with a paralytic virus as they are during parasitic mite syndrome^34,37^. We therefore determined the abundance and prevalence of three virus know to be associated with infestations of *V. destructor* at three different apiaries; two diagnosed with EFB disease and one diagnosed with putative Varroosis or PMS (File S4). Melt curves of qPCR products correspond to the RNA positive controls confirming an uncontaminated PCR product. For all three quantified virus, samples from Spring Valley and Butler were often undetectable with Cq values > 40. Therefore, as a conservative measure, we chose the values recorded from non-symptomatic (healthy) hive samples in the Hull apiary (mean Cq = 29.92) to calibrate fold differences in the data set, and perform statistics. We found that all three virus were significantly elevated in the putative PMS samples from the Hull apiary, but ABPV was overwhelmingly the dominant virus, accounting for > 99.7% of the virus copies (Fig. 5). At the putative PMS apiary (Hull) the levels of ABPV were many fold greater than that returned for the EFB apiaries (File S4, F_2,134_ = 86.5, p < 0.00001).

Regardless of *M. plutonius* levels, ABPV and KBV were mostly undetectable at the EFB diseased apiaries Spring Valley and Butler. In contrast, DWV was prevalent at low abundance across all three tested apiaries. DWV was significantly elevated in symptomatic larvae from both Hull and Butler apiaries (F_8,128_ = 7.7, p < 0.0001), while KBV was also slightly but significantly elevated in symptomatic larvae at the Hull apiary (F = 6.4, p < 0.0001), indicating opportunism following, or concurrent with, primary infection with either ABPV or *M. plutonius*. Neither KBV nor DWV were elevated in asymptomatic larvae. In contrast, asymptomatic larval instars within the symptomatic PMS colony at the Hull apiary contained significantly elevated ABPV copy number relative to healthy larvae from other apiaries (Fig. 5). Beyond this, our sampling design captured significantly elevated ABPV levels in asymptomatic 3rd, 4th and 5th larval instars from an asymptomatic colony at the Hull apiary (File S4, Steel–Dwass; q = 3.1, p < 0.05).

## Discussion

We employed a certified state apiarist to perform a survey of brood disease in Illinois, sampling disease states identified in the field as EFB or EFB-like according to larval symptomology. From each apiary, we sampled symptomatic and asymptomatic colonies, capturing brood disease microbiomes as they relate to visual identification and photographic documentation (File S1). With our experimental design, we aimed to describe the distribution/heterogeneity of microbial diversity throughout larval development and disease progression (Fig. 1). To accomplish these goals we characterized the larval microbiota from an apiary in Arizona with no present or recent brood disease. From the symptomatic apiaries in Illinois, we characterized two major disease states that afflict honey bee larvae, one caused by *M. plutonius* (European Foulbrood, EFB), the other caused by Parasitic Mite Syndrome (Varroosis) and Acute Bee Paralysis Virus (PMS-ABPV). Below we discuss the general nature of the results, healthy larval microbiotas, EFB-associated microbiotas, and a novel disease microbiota associated with the symptomology of PMS-ABPV.

### The healthy larval microbiota

From the disease free apiary, the microbiota generally increased in size with larval development (Fig. 2), and varied by developmental stage. Consistent with the occurrence of multiple pathogen species in the microbiota of early stage larvae, we suggest that larval development may involve immune training as in other organisms^68^. In older larvae, the healthy microbiota is dominated by *Bo. apis* and/or *A. kunkeei*, both prevalent core gut bacteria of reproductive queens^64,69,70^. Over 95% of the queen and worker mouthparts and anterior alimentary tract of queens, classify as these two oxygen tolerant species. Both bacteria are associated with decreased abundance of honey bee-specific disease, and likely provide protection from many aerobic opportunists including bacteria, microsporidia, and fungi, omnipresent throughout the social resource environment^31,71,72^. Not associated with disease, but occurring somewhat opportunistically in six of our seven apiary samples, Lachnospiraceae (*Clostridia* spp.) is seemingly common to the larval gut environment, but requires formal description. Similar to *Bifidobacterium* and *Gilliamella* in the hindguts of adult honey bees^73^, these bacteria ferment a diverse group of plant polysaccharides and their constituent monosaccharides to short-chain fatty acids^74^. It appears that this aerotolerant larval microbiota is ancestral to *Apis,* because *Apis florea* and *Apis ceranae* larvae possess highly similar species including *Bombella, A. kunkeei, Gilliamella*, *Clostridia*, and the *Apis mellifera* EFB pathogen, *M. plutonius*^61,62^.

Common in healthy larvae, *Apilactobacillus kunkeei* is a lactic acid bacteria adapted to fructose-rich niches like honey and royal jelly^75,76^. A previous study reported that the culture supernatant from an *A. kunkeei* isolate inhibited growth of *M. plutonius*^77^. More recently it was determined that *A. kunkeei* produces kunkecin, a newly described Bacteriocin with a narrow antibacterial spectrum, and high antibacterial activity against *M. plutonius*, indicative of long term co-evolution in the same niche^31^. The larval niche includes the antimicrobials expressed in royal jelly, but *Bombella apis* flourishes in this environment^17,59^, with a genome optimized for rapid energy production^78^. Worker bees secrete glucose oxidase into royal jelly and convert glucose into a lactone of D-gluconic acid and hydrogen peroxide defining one antibiotic mechanism of royal jelly^79^. However, *B. apis* can further oxidize gluconolactone into gluconate, which is then shunted into the ED pathway^78^ potentially facilitating its rapid growth in royal jelly and larvae.

Many of the species found in larvae are known to reproduce in royal jelly such that variations in the quality of royal jelly may influence microbial proliferation^80,81^. Like other resident bacteria, *M. plutonius* has the genes to survive in pollen, honey and royal jelly and is often detected when sequencing adult guts, suggesting that it is somewhat ubiquitous in *Apis mellifera*, surviving at low prevalence and abundance when no symptoms are present (Fig. 3). As suggested elsewhere, colonies that have been stressed or weakened nutritionally may produce less than adequate royal jelly with more water and less antibiotic activity permitting greater microbial growth. Over the first two instars, the jelly fed to larvae is high in protein and lipid content, but the character of jelly shifts at the third instar, and becomes increasingly sugar rich^80^. A clear contributor to disease, poor nutrition may reduce the antimicrobial character of royal jelly^82^. Given the early supply of potent royal jelly, the larval gut environment may more accurately reflect what can survive in the early nourishment medium more than it does direct bacterial competition^81^. While *Bombella apis* thrives in royal jelly, *M. plutonius* can survive long enough to populate the larval gut, but this ability varies by strain^6,59^.

### Asymptomatic larval microbiota differs by apiary

In colonies with clinical EFB symptoms, adult worker bees in contact with the brood generally possess greater than 5 × 10^4^ CFUs of *M. plutonius* per bee^83^. Workers drifting from one colony to another is suspected to be the major mode of EFB transmission, such that during an apiary outbreak of EFB, nearby asymptomatic hives may also harbor an elevated load of *M. plutonius*^32,60^. Additionally or alternatively, *M. plutonius* is ubiquitous, and a shift in environmental conditions encouraged its reproduction at the apiary level. Consistent with either prediction, the bacterial abundance and taxonomy of asymptomatic larvae differed when comparing the disease free apiary to apiaries experiencing active disease (Fig. 3, File S3). From both environments, we used the same standard curve to estimate bacterial load such that the differences are unlikely methodological. However the comparison is not exact; the disease-free, known-age data set was sampled strictly every 24 h following egg hatch resulting in the youngest and smallest instars based on weight. For example, our 104 h-old larval sample approximating fourth instar larvae weighed an average of 42 mg, but larvae entering pupation (day 7 after egg hatch) are typically > 150 milligrams^42^. To control for variation in size, we compared 80 and 104-h-old larvae with 3rd and 4th instar larvae (Fig. 2), and found similar bacterial load for asymptomatic colonies at two of six diseased apiaries; Hardin and Hull. Asymptomatic bacterial load was significantly elevated at the other four apiaries, and third instars were host to many of the same disease-associated bacteria seen in early control larvae from the disease free apiary (Fig. 4).

However, disease bacteria dominated only three of 24 asymptomatic fifth instar larvae sampled from asymptomatic colonies in Illinois, and either *B. apis* or *A. kunkeei* dominated the remainder. This pattern suggests that older larvae from the same colony were exposed to various pathogens but cleared them as younger larvae. Many factors could contribute to this colony-level pattern including the nutritional environment (royal jelly quality), host genotype, and pathogen virulence^27,60,81,84^. In contrast, we found that most asymptomatic larvae in an EFB diseased hive possess significantly elevated levels of *M. plutonius*, indicating rapid in-hive transmission facilitated by the nursing activity and worker secretory glands/mouthparts^85^.

### European foulbrood microbiota

We found that *M. plutonius* differed in abundance and prevalence when comparing EFB positive apiaries, perhaps an indication of sampling, host susceptibility or pathogen virulence. Given the variation in key pathogen genes, toxins and virulence plasmids, ability to grow aerobically and form biofilms, *M. plutonious* demonstrates a broad range of virulence^27,29,86^. Low virulence strains of *M. plutonius* may typically act as social symbionts, only becoming opportunistic when the host is stressed. The presence of bacteriocins found in all typical strains of *M. plutonius* may indicate competition with the native microbiome, and specifically the larval gut microbiome during EFB outbreaks^27^.

Our use of BEExact to classify OTUs at the lowest possible taxonomic level provides the most accurate picture to date of the bacteria associated with EFB disease progression. We found that *Melissococcus plutonius* (EFB) co-occurred with significantly different microbial taxa and community structures by sampled apiary (Fig. 4A–E). Past work has suggested that EFB disease is involves “helper” bacteria acting as either saprophytes or secondary invaders^33,58,81,85^. Many bacteria species from the adult honey bee microbiota form metabolic partnerships with other species and such organization likely applies to various disease pathologies^87^. Based on a literature search, potential helper species associated with EFB disease may include *Paenibacillus alvei, Enterococcus faecalis, Brevibacillus laterosporus and Achromobacter eurydice*^33,58,60^. It was recently deduced that *A. euyridice,* was most likely *Apilactobacillus kunkeei*^58^, an abundant species in many larval guts (Figs. 2, 3), both healthy and diseased^59^. Our high throughput sequencing method did not return *Brevibacillus* with any frequency or prevalence in EFB diseased larvae (Fig. 4, File S3). *P. alvei* occurred at one of the five EFB apiaries in symptomatic larvae and those with advanced disease. At the other four apiaries with confirmed EFB, *Enterococcus faecalis*^88^ was prevalent and abundant, showing a pattern of co-infection at the Hardin apiary (Fig. 4C, File S3).

Based on Wilcoxon rank sum tests, we recorded nine abundant bacteria that increased significantly with *M. plutonius* in diseased larvae; *Enterococcus faecalis*, *Frishella perrara*, *Lactobacillus apis*, *Fructobacillus fructosus*, *Lonsdalea iberica, G. apicola, Bombella apis, Bombella* spp*., S. alvi,* and *Bifidobacterium*. In agreement with culture based results^11^, gram positive bacteria, primarily *L. kunkeei, F. fructosus* and *L. firm5* became more abundant with advanced EFB disease in a site-specific manner (Fig. 4A–E). *Frischella perrara* may have been a secondary invader at the Hardin apiary based on occurrence patterns (Fig. 4C). *Frischella* overgrowth causes scab formation in the adult pylorus^89^, and is associated with poor nutrition and gut dysbiosis in adult workers^12^. At three of the five EFB positive apiaries (Fig. 4B,C,E), *A. kunkeei* was positively associated with *M. plutonius*, occurring throughout EFB disease progression, and often attained high numbers in larvae with advanced disease symptomology. Based on the genomic data and microbiome size, these disease microbiotas likely reflect bacterial competition refined over many millennia^27,31^.

### A microbiota of Varroosis (parasitic mite syndrome)

The Hull apiary likely suffered from Varroosis. To place our molecular metrics in context, we discuss the following colony level observations derived from the apiary inspector or from photographs of the diseased frames containing symptomatic larvae (File S1). The diseased colony at the Hull apiary had a very low population size, and a queen with a reduced laying pattern. The frames were characterized by brood cappings that contained holes or perforations. From the photograph of the diseased frame, the beginnings of two queen cells are evident, suggesting that a dwindling colony was attempting to replace a substandard queen. Many photographs captured the ectoparasitic mite *V. destructor* outside of a brood cell, suggesting high mite loads (File S1). The symptomology provided by the apiary inspector agrees with the photographs, uniquely describing larvae with extremely elevated ABPV levels as either “melting or deflated”. These descriptors were not applied to the EFB symptomology in this study, but are used to describe both PMS and IBDS^37^. The relationship between the melting /deflated symptomology and extremely elevated ABPV (> 10^7^) was 100% at the Hull apiary, although this sample size was represented by only thirteen overtly diseased larvae (Fig. 4F, File S4).

Considered the most destructive disease of honey bees worldwide, Varroosis may involve many different virus^35^. Based on the combined metrics and observations, we conclude that Varroosis caused the disease state at the Hull apiary in combination with acute bee paralysis virus (Fig. 5). Also referred to as parasitic mite syndrome (PMS), this colony-level disease state is defined by high mite loads and infection with paralytic viruses transmitted by *V. destructor* including those we tested among others^38^. We found that the relative and absolute levels of ABPV throughout larval development was highly consistent with an acute disease state (Fig. 4F, Fig. 5). The pattern of ABPV occurrence and prevalence indicates that ABPV is highly virulent and transmissible at the Hull apiary, even in asymptomatic hives, where we found elevated levels in young larvae (Fig. 5). As part of their strict life history, *V. destructor* are hardwired to feed on late fifth instar larvae/early pupae^36^, such that ABPV transmission to young larvae must occur via a different route. The prevalence of ABPV in adults is highly associated with the abundance of the *V. destructor* mite^39^, suggesting that the disease first manifests in adults that have been parasitized by *V. destructor*, and is then transferred to larvae. Based on one study, ABPV requires only 10^2^ virus copies to attain virulence when injected into the adult hemolymph (*V. destructor* makes a hole in the abdomen), but 10^8^ when consumed^40^. ABPV then accumulates in adult tissues of the brain, fatbody and hypopharyngeal glands^90^, and is secreted directly into the larval food as suggested by earlier work^91^.

We found that the bacterial microbiota associated with ABPV-PMS did not resemble the EFB disease microbiota (Fig. 4A–F, File S3). The PMS/APBV disease microbiota showed significant increases in *Serratia marcescens;* a demonstrated pathogen of adult and larval honey bees^92,93^, and *S. alvi, G. apicola, and F. perrara*; core ileum/pylorus bacteria of adults^94^. Moreover, *S. alvi* and *S. marcesens* were strongly correlated following a log transformation of bacterial abundance suggesting synergistic co-existence (Adj Rsq = 0.59, F = 31.1, p < 0.0001). Bacteria are also vectored by the *V. destructor* mite including *S. marcescens* and *E. faecalis,* and bacteria native to the honey bee worker ileum^95,96^. While the effects of *S. marcescens* in honey bees are becoming evident^10,13,93,97^, this bacterium is a known secondary invader following viral and other infections, and can be abundant in *V. destructor* mites and adult bees sampled from stressful overwintering conditions^93^. In a recent paper, *S. marcescens* is proposed as a widespread opportunistic pathogen of adult honey bees that may be often go undetected, but is highly virulent in adults following exposure to the antibiotic tetracycline^14^.

Our results further suggest that the microbiome composition of PMS larvae in this study was fashioned in part by beekeeper-applied antibiotics (File S1). According to the Hull apiary beekeeper, the PMS colony was treated with Tetra Bee (oxytetracycline) a couple weeks prior to sampling, but the disease state was unaffected. Consistent with long-term effects of this antibiotic^98^, both healthy and disease microbiomes from this location showed a significant deficit of gram-positive bacteria relative to the other apiaries (File S3). Gram-negative species known to carry antibiotic resistance genes were the only bacteria found blooming in diseased larvae^99^. This suggests a scenario wherein the overtreatment with antibiotics interfered with normal microbiome function, rendering the host more susceptible to opportunistic and antibiotic resistant microbes including DWV and KBV, both significantly elevated in larvae with advanced disease (File S4).

## Conclusion

European foulbrood and Varroosis are associated with substantial colony loss. The application of BEExact to classify OTUs at the lowest possible taxonomic level provides the most accurate picture to date of the bacteria associated with larval microbial succession and disease progression. This work characterizing healthy and diseased larvae contributes to a holistic understanding of the microbiome in *Apis mellifera*. We conclude that EFB manifests in a variety of ways, and our sampling strategy within an apiary provides insight into disease progression. Asymptomatic samples varied by *B. apis* and *A. kunkeei* abundance and taxonomy, perhaps affecting the mode of disease progression. While bacteria and virus are typically studied in isolation, here we present a simultaneous assessment of both factors to further our understanding of opportunistic disease progression in the highly social honey bee. Future investigations of Varroosis/PMS/IBDS in honey bees should apply multiplexed detection of virus^100^.

## Supplementary Information

Supplementary Information 1.

Supplementary Information 2.

Supplementary Information 3.

Supplementary Information 4.

## Data Availability

The raw 16S rRNA gene sequence reads were deposited with the National Center for Biotechnology Information, BioProject Accession: PRJNA897937 http://www.ncbi.nlm.nih.gov/bioproject/897937.
